# Efficient Blue to Red Afterglow Tuning in a Binary Nanocomposite Plastic Film

**DOI:** 10.3390/nano8040260

**Published:** 2018-04-19

**Authors:** Yan Xia, Huase Ou, Wanbin Li, Gang Han, Zhanjun Li

**Affiliations:** 1 Guangdong Key Laboratory of Environmental Pollution and Health, School of Environment, Jinan University, Guangzhou 510632, China; yanxiajnu@sina.com (Y.X.); ouhuase@126.com (H.O.); gandeylin@126.com (W.L.); 2 Department of Biochemistry and Molecular Pharmacology, University of Massachusetts Medical School, Worcester, MA 01605, USA; gang.han@umassmed.edu

**Keywords:** persistent luminescence, afterglow, spectral tuning, nanophosphor, nanocomposite

## Abstract

Colorful spectra are important for the diverse applications of persistent phosphors. A color conversion concept is developed to obtain abundant persistent luminescence color by mining capacities of known persistent phosphors with the most efficient persistent properties. Here, SiO_2_/Sr_2_MgSi_2_O_7_:Eu,Dy nanoparticles are chosen as a blue persistent luminescence donor nanophosphor, while ultrafine CaAlSiN_3_:Eu is utilized as a red conversion phosphor to tune the persistent luminescence spectra from blue to red. The red afterglow emission can persist for more than 5 h. The decay of the red afterglow follows nearly the same kinetics as that of the blue one. Continuous color tuning can be successfully obtained by simply changing the mass ratio of the donor/conversion phosphor pair. This color conversion strategy may be significant in indicating numerous persistent/conversion nanocomposites or nanostructures and advance the development of persistent phosphors in diverse fields which need colorful spectral properties.

## 1. Introduction

The emission of persistent phosphors can persist for a long period after excitation ceases. Persistent luminescence and persistent phosphors are arousing increasing interests and have found abundant applications in night vision materials, security signals, art painting, mechanical stress detection, high energy particle detection, optical information storage, catalysis, analysis, time-resolved bioimaging, etc. [1,2,3]. Apparently, these applications rely on the synthesis of persistent phosphors with abundant spectral properties. Many new phosphors have been reported, usually by developing new matrix and/or new dopants. For example, Ca_0.2_Mg_0.9_Zn_0.9_Si_2_O_6_:Eu,Dy,Mn is the first studied persistent phosphor in persistent luminescence imaging [4,5]. Cr-doped gallium oxide related phosphors represent the current dominating ones [6,7,8,9,10,11,12]. Novel Cr/Ga-free persistent phosphors are being explored such as Zn_3_Ga_2_Ge_2_O_10_:Ni^2+^ [13], LaAlO_3_:Mn^4+^,Ge^4+^ [14], BaZrSi_3_O_9_: Eu^2+^, Pr^3+^ [15], KGaGeO_4_:Bi^3+^ [16], semiconducting polymer [17], metal organic framework [18]. To date, Sr_2_MgSi_2_O_7_:Eu^2+^,Dy^3+^ still represents one of the most efficient commercial ones [19,20]. However, there is still not a red persistent phosphor with afterglow properties close to the commercialized blue or green ones. A novel strategy is needed to realize efficient red persistent luminescence to facilitate the practical applications of persistent phosphors.

Color conversion has been widely applied in the field of LED lighting to obtain white light [21]. Generally, a blue semiconductor lighting chip and color conversion phosphors are mixed together. Then, the color conversion phosphors absorb part of the blue light and emit green, yellow, and/or red light to generate abundant visible colors [22,23,24,25]. Yuhua Wang’s group successfully introduced color conversion to generate white persistent luminescence by using CaAl_2_O_4_:Eu^2+^,Nd^3+^ bulk material as blue persistent phosphor and Y_3_Al_5_O_12_:Ce^3+^ as conversion phosphor [26]. As bulk raw materials were used, the amount of conversion phosphor has to be as much as 50% which will decrease the emission intensity and lead to heterogeneity. Using nanophosphors may generate more efficient color conversion or energy transfer. Inspired by these previous works, we propose that persistent spectral tuning can be realized efficiently by exploring known persistent nanophosphors via color conversion. Here, a blue persistent nanophosphor, SiO_2_/Sr_2_MgSi_2_O_7_:Eu^2+^,Dy^3+^ nanoparticle (SMS), is chosen as a blue persistent energy donor as Sr_2_MgSi_2_O_7_:Eu^2+^,Dy^3+^ is currently one of the most efficient blue persistent phosphors [27,28] and can be easily synthesized in nanoscale [19,20,21,22,23,24,25,26,27,28,29]. CaAlSiN_3_:Eu^2+^ (CASN), which is one of the most efficient red color conversion phosphors in LED industry, is used as a color conversion phosphor [30,31,32]. SMS and CASN ultrafine particles are used to obtain a uniform binary persistent luminescence nanocomposite system. The nanocomposite is buried into polymethyl methacrylate (PMMA) plastic film to make the particles stay close to each other and thus to ensure efficient conversion efficiency.

## 2. Experimental

### 2.1. Synthesis of SMS

The synthesis of SMS was according to our reported template method with modifications [33,34]. Briefly, 0.1273 mol of Mg(NO_3_)_2_·6H_2_O, 0.0667 mol of Sr(NO_3_)_2_, 0.01 mol of HBO_3_, 0.002 mol of Eu(NO_3_)_3_, and 0.004 mol of Dy(NO_3_)_3_ were dissolved in deionized water with a total volume of 100 mL according to the formula of Sr_2_MgSi_2_O_7_:Eu_0.01_,Dy_0.02_, forming a precursor solution of SMS. The total molar concentration of metal ions was controlled to be 2 M. Five percent (molar ratio vs. metal ions) of HBO_3_ was used as flux. Ten grams of mesoporous silica nanoparticles (100 nm–150 nm) were mixed with 100 mL of SMS precursor solution, stirred for 1 h, centrifuged, and dried at 105 °C for 2 h. The dried precursor was firstly annealed at 800 °C for 2 h. The product was ground using a motor and pestle. SMS nanoparticles were finally obtained after being annealed at 1000 °C for 5 min at a heating rate of 10 °C/min. Both of the heating procedures were carried out at a reductive (H_2_/Ar, 10%) atmosphere.

### 2.2. Synthesis of CASN

CASN was prepared by a reported solid state method followed by a ball milling process [31].

### 2.3. SMS/CASN Film Preparation

SMS/CASN composite with diverse mass ratio was homogenized by ball milling. Then, 0.1 g of the composite was mixed with 1 mL of PMMA/dichloroform (20%) solution. The mass content of PMMA in the film is ca. 66.7 wt %. The mixture was stirred at room temperature to let dichloroform evaporate and form a slurry, which was poured into a round mould with a diameter of ~2 cm and a thickness of 1 mm. A SMS/CASN plastic film was obtained after aging the mould in open air at room temperature (~20 °C) overnight.

### 2.4. Characterization

The X-ray diffraction pattern was acquired by using a powder diffractometer with Cu Kα radiation (λ = 1.5418 Å) (D2 PHASER, AXS, Karlsruhe, Germany). The photoluminescence and persistent luminescence spectra were obtained by using a fluorophotometer (Lumina, ThermoFisher, Waltham, MA, USA). The afterglow spectra were activated by using a violet LED torch (395 nm, 9 w) and acquired at 10 min after the stop of excitation. The microstructures of the phosphors were observed by using a field emission scanning electron microscope with an accelerating voltage of 5 kV (S-4800, Hitachi, Tokyo, Japan). Three-dimensional luminescent imaging of the nanocomposite film samples was conducted using a Bio-Rad gel imaging system.

## 3. Results and Discussion

**Figure 1:**
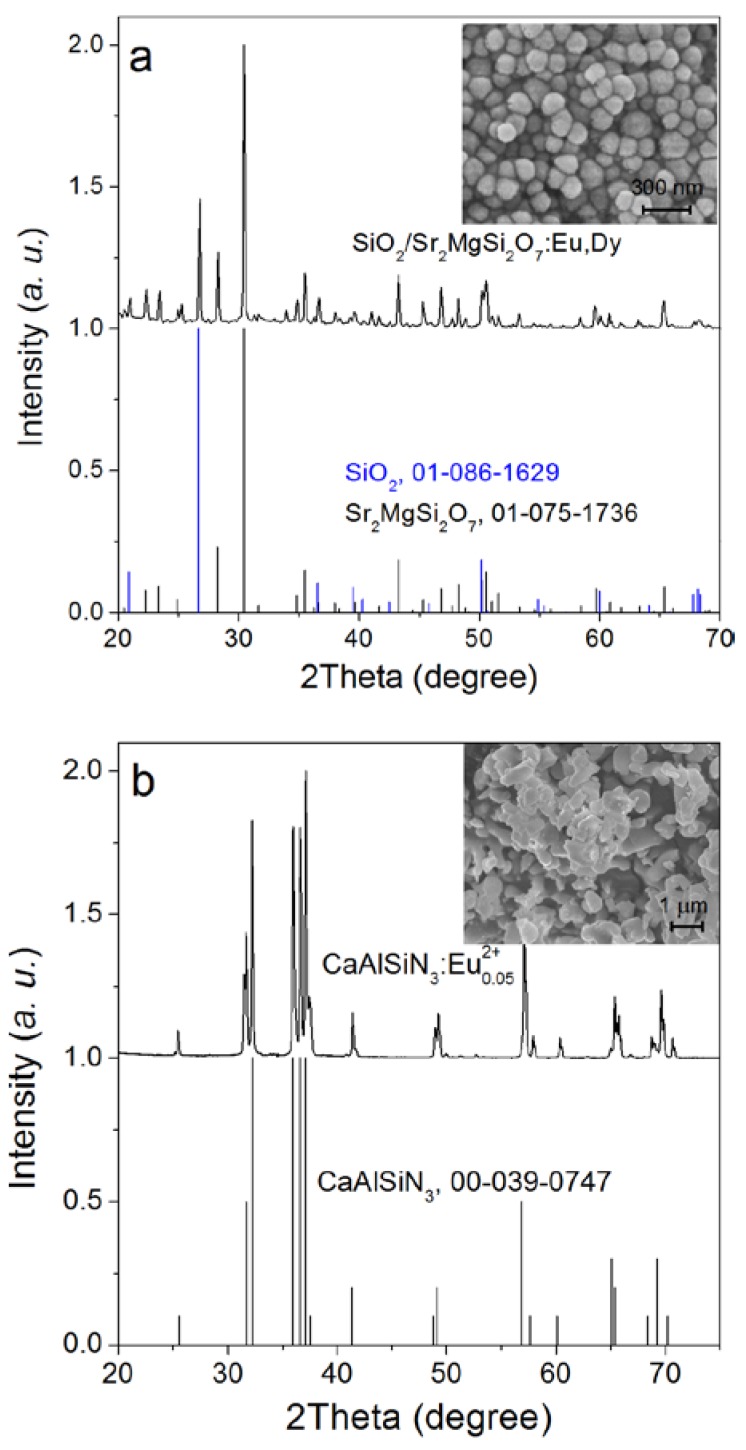
X-ray diffraction patterns and scanning electron microscopy images of the as-synthesized SMS (SiO_2_/Sr_2_MgSi_2_O_7_:Eu^2+^,Dy^3+^ nanoparticle) (**a**) and CASN (CaAlSiN_3_:Eu^2+^) (**b**) ultrafine particles.

Alkaline earth silicate nanoparticles can be synthesized by using mesoporous silica nanoparticles as morphology-controlling templates, forming a nanocomposite of quartz and persistent phosphors [33,34]. As indicated in Figure 1a, the diffraction pattern of the as-synthesized SMS corresponds well with that of tetragonal Sr_2_MgSi_2_O_7_ (JCPDS card no. 01-075-1736) and hexaganol SiO_2_ (JCPDS card no. 01-086-1629). No apparent impurity diffraction peaks can be observed, indicating the successful doping of Eu and Dy into Sr_2_MgSi_2_O_7_ lattice. The size of SMS particles ranges from ~80 nm to ~150 nm. The as-synthesized CASN possesses an orthorhombic crystal structure with diffraction pattern corresponding with JCPDS card no. 00-039-0747 (Figure 1b). The as-synthesized CASN has a size ranging from ~200 nm to ~2 μm. Both SMS and CASN samples have relatively small sizes which is smaller than most of the commercial LED conversion phosphors (~10 μm). Thus, uniform homogenization and efficient color conversion may be possible.

**Figure 2:**
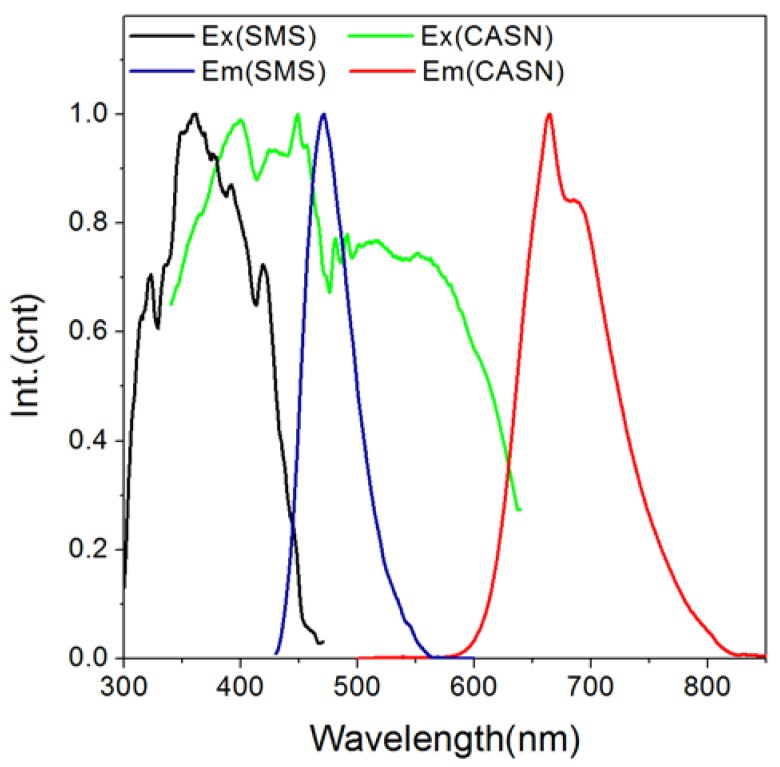
Photoluminescence excitation/emission spectra of the as-synthesized SMS and CASN nanoparticles, respectively. The excitation spectra of SMS and CASN were monitored at 470 nm and 665 nm, respectively. The emission spectra of SMS and CASN were excited at 400 nm and 470 nm, respectively.

**Figure 3:**
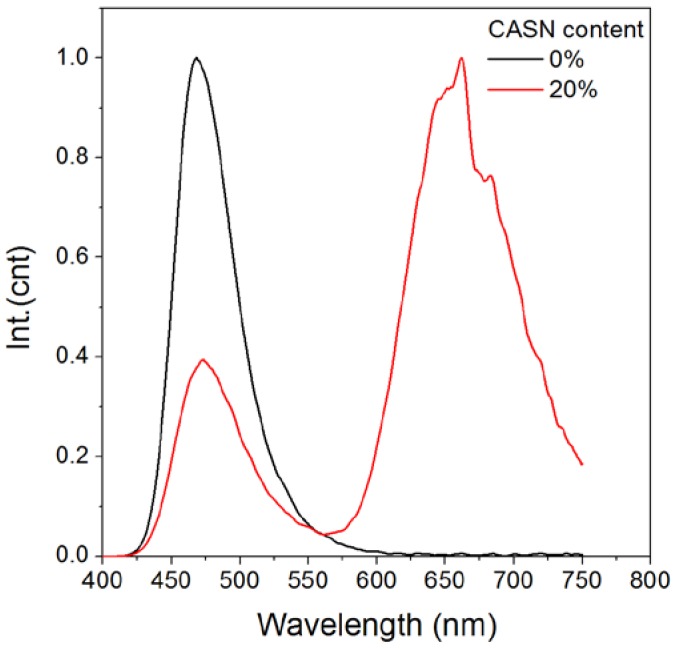
Afterglow emission of SMS and CASN/SMS nanocomposite PMMA (polymethyl methacrylate) film (20% of CASN by weight). Samples are excited by using a violet LED torch (395 nm, 9 w).

In order to establish an efficient color conversion luminescence system, an overlap between the donor/acceptor is a prerequisite [35,36,37]. The photoluminescence excitation/emission spectra of CASN in Figure 2 indicate that CASN phosphor can be efficiently excited by wide spectrum extended from UV to 600 nm and emit red light peaking at ~660 nm. Notably, its excitation spectrum overlays well with the persistent luminescence spectrum of SMS from 430 nm to 550 nm with a peak at ~470 nm. Thus, CASN can serve as a possible efficient conversion phosphor for SMS. We prepared a nanocomposite of SMS and CASN which contains 20% (by weight) of CASN mixed by ball milling and then buried into PMMA film. After excited by a blue LED torch, the as-prepared SMS/CASN/PMMA film possessed bright red afterglow persistent luminescence in the dark. Its afterglow emission spectrum possessed a weak blue peak at ~470 nm and an intense red one at ~660 nm (Figure 3). The blue peak comes from the typical afterglow emission of Eu^2+^ in SMS. The red peak comes from the photoluminescence of Eu^2+^ in CASN. The high red/blue ratio indicates a successful color conversion process.

**Figure 4:**
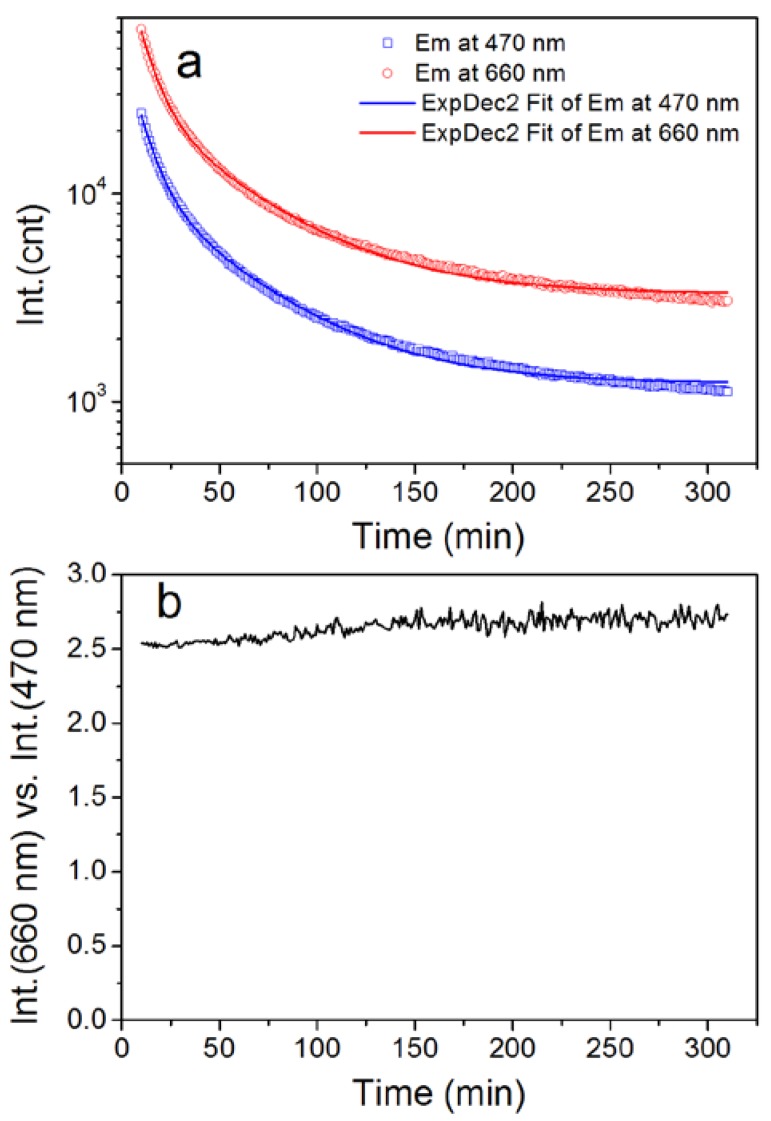
Afterglow decay of the CASN/SMS (1/4, by weight) nanocomposite film: (**a**) Emission at 470 nm/660 nm; (**b**) red/blue intensity ratio, Int.(660 nm/Int.(470 nm), within 300 min. A 10 min delay was used because of the intense luminescence intensity at the start of the decay process and the over range protection of the fluorophotometer.

The afterglow emission of the CASN/SMS nanocomposite PMMA film can be detected for more than 5 h at 470 nm/660 nm, as shown in Figure 4a. Its decay kinetics can be fitted successfully by a ExpDec2 function, y = A_1_ · exp(−x/t_1_) + A_2_ · exp(−x/t_2_) + y_0_, using origin software, as shown in Table 1. The similar values of t_1_ and t_2_ in the fitted decay curves of 470 nm and 660 nm indicate that the afterglow emissions at 470 nm and 660 nm follow similar decay kinetics and can ensure stable color during its decay process. The afterglow intensity ratio of Int.(660 nm) vs. Int.(470 nm) fluctuates at a narrow range from 2.5 to 2.7 within 5 h during the whole afterglow process, which also indicates stable spectral color of the afterglow emission. Both of the two decay curves include a fast decay period and a relatively slow one. The fast decay period in the beginning comes from the release of the stored energy in shallow traps, while the slow one originates from the release of the stored energy in deeper energy traps. Similar results have been revealed in previous reports about afterglow mechanisms of Sr_2_MgSi_2_O_7_:Eu,Dy [38,39,40].

**Figure 5:**
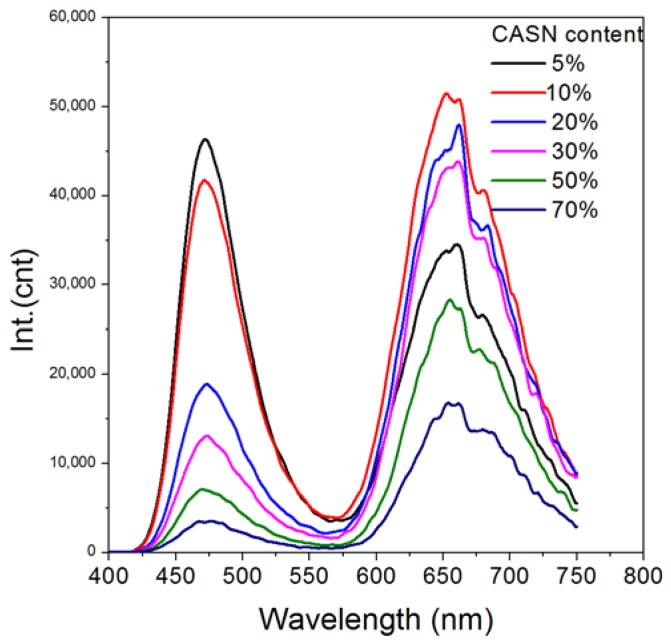
Afterglow emission spectra of CASN/SMS nanocomposite films with diverse CASN content. Samples are excited by using a violet LED torch (395 nm, 9 w).

**Figure 6:**
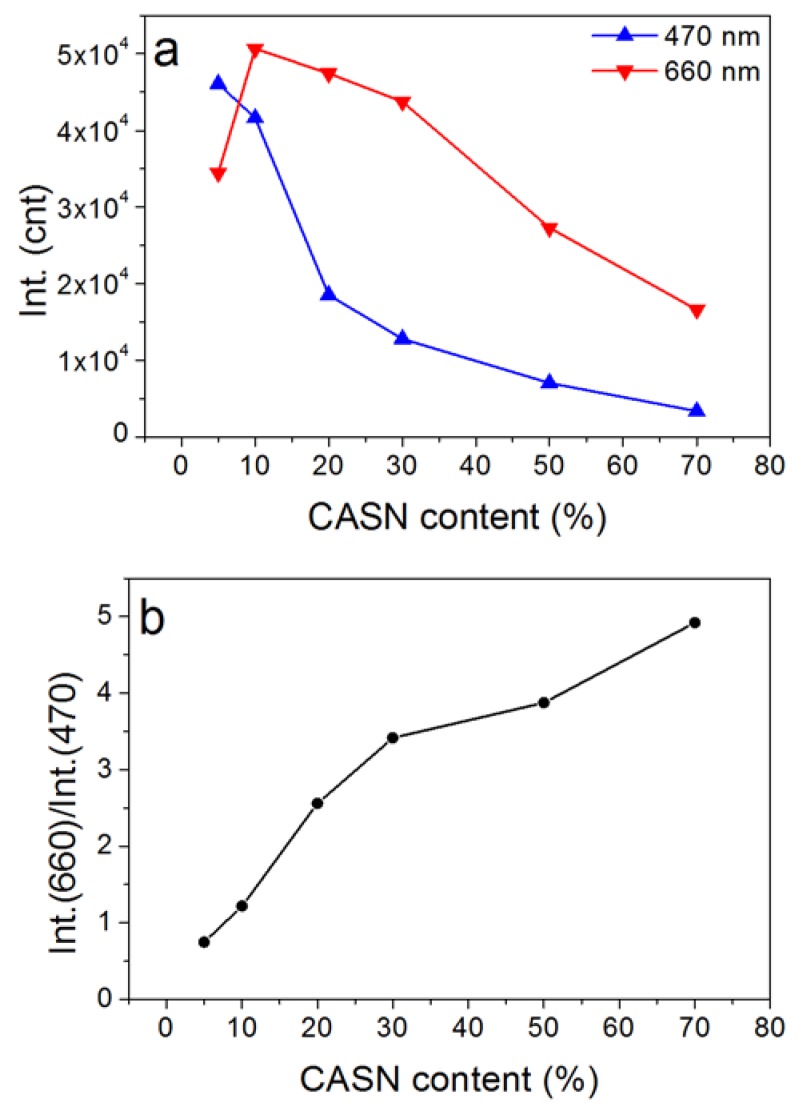
Influence of CASN content on the intensity (**a**) and red/blue emission ratios (**b**) (Int.(660 nm) vs. Int.(470 nm)) of the nanocomposite films.

In order to study the influence of mass ratio to spectral tuning, CASN/SMS nanocomposite films with diverse mass ratios were prepared. Their afterglow emission spectra (Figure 5) show the blue persistent luminescence of SMS at ~470 nm. A new peak appears at ~660 nm, which can be assigned to the fluorescence emission of CASN excited by the persistent luminescence of SMS. As indicated in Figure 6, the blue peak decreases because of the decrease of the mass of SMS in the film and the absorption of CASN along with the increasing amount of CASN in the film. Higher red/blue ratio (Int.(660 nm) vs. Int.(470 nm)), which means redder visual color, can be obtained if more CASN is used. Yet, the total persistent luminescence intensity will decrease along with an increasing CASN content due to less SMS exists. The optimal mass ratio of CASN vs. (CASN + SMS) is found to be 10% because of its maximal emission at 660 nm in all the film samples which has a red/blue ratio of ~1.2. Notably, a relatively small amount of conversion phosphor (~10%) is needed to generate a successful and apparent persistent luminescence color conversion process, possibly because of the ultrafine particle sizes of SMS and CASN which provides uniform homogenization. Regarding energy transfer between particles, a small distance is generally favorable. Using SMS nanoparticles may possibly limit the distance between the persistent luminescence energy donor and conversion phosphor within the range of ca. 100 nm and realize more efficient persistent luminescence color conversion. According to a recent publication, 50% or more conversion phosphors will be needed to realize apparent color conversion in the case of bulk phosphors [26]. Thus, nanocomposites or nanostructures may be a possible efficient new way to explore abundant persistent luminescence spectra by taking full advantage of existing persistent phosphors and conversion phosphors.

**Figure 7:**
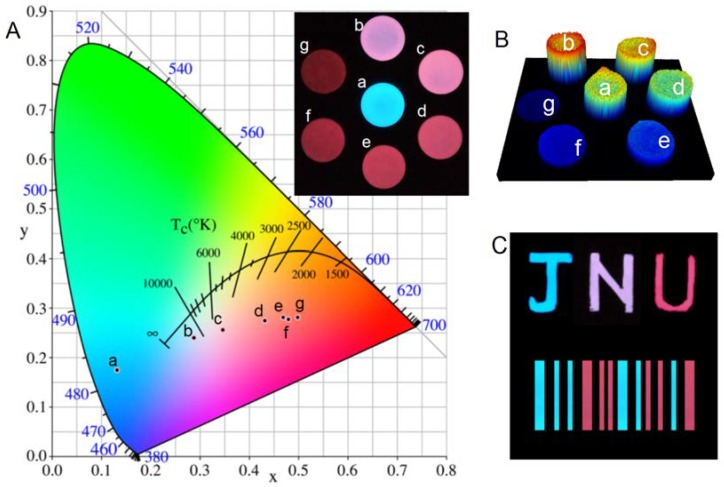
(**A**) CIE (Commission Internationale de L'Eclairage) chromaticity diagram and digital pictures of the multicolor SMS/CASN nanocomposite film samples with diverse CASN contents. (**a**) 0%, (**b**) 5%, (**c**) 10%, (**d**) 20%, (**e**) 30%, (**f**) 50%, and (**g**) 70%. The digital pictures were taken by using a SLR (single-lens reflex) camera after the stop of LED excitation (395 nm) for 5 min; (**B**) Three-dimensional luminescent imaging of the nanocomposite film samples (diameter 2 cm, thickness ~0.2 mm). The luminescence intensity is expressed by false color and height; (**C**) Potential applications of multicolor afterglow nanocomposite. The letters J, N, and U are painted by using nanocomposites with CASN contents of 0%, 5%, and 20%, respectively. The bar code is painted by using nanocomposites with CASN contents of 0% (blue) and 20% (red).

The visible color of the samples can be gradually tuned from blue (SMS) to deep red when the content of CASN is increased from 0% to 70% (Figure 7). Regarding to red persistent luminescence, Y_2_O_2_S:Eu^3+^,Mg^2+^,Ti^4+^ and CaS:Eu^2+^,Tm^3+^ are representative commercial persistent phosphors. However, Y_2_O_2_S:Eu^3+^,Mg^2+^,Ti^4+^ can only be excited efficiently by UV or violet light because of the spin-forbidden 4*f*-4*f* transition of Eu^3+^, which means that it can’t be excited efficiently by room light, which will hinder its practical applications [41]. CaS:Eu^2+^,Tm^3+^ can be excited efficiently by room light but suffers from fast decay and poor water resistance [42]. SMS/CASN composite film possesses bright red persistent luminescence after blue light excitation, which can be seen for more than 5 h in the dark by naked eyes. 3-D luminescence imaging was performed in a bio-rad gel imaging system to study the luminescence homogeneity of the composite films, in which the intensity of each pixel is expressed in false rainbow color (blue to red colors represent intensities from weak to strong) and height. The result in Figure 7B indicates that the use of nanoscale or ultrafine particles can provide good spatial color homogeneity. The multicolor afterglow nanocomposite may be painted into colorful afterglow patterns like afterglow logo and bar code as shown in Figure 7C. Thus, this color conversion strategy may find potential applications such as anti-fake painting, information encryption, safety identification, and in energy saving lighting strategies.

## 4. Conclusions

SMS, an efficient blue persistent nanophosphor, can serve as an efficient persistent nanometer energy donor to excite CASN, a red emitting phosphor without persistent properties. The red afterglow emission can persist for more than 5 h. The decay of the red afterglow follows nearly the same kinetics as that of the blue one. The continuous color tuning afterglow nanocomposite material or device can be obtained by simply changing the mass ratio of the donor persistent nanophosphor and conversion phosphor. Although this work only extends the persistent luminescence spectra from blue to deep red, we believe that near infrared persistent luminescence may also be realized by using a similar strategy. In addition, this work may be significant in indicating possible efficient persistent luminescence nanostructures and nanodevices in the future. This work may further advance the development of colorful persistent phosphors in diverse fields.

## Figures and Tables

**Table 1:** Decay kinetic parameters of the afterglow emission of CASN/SMS (CASN content, 20 wt %) nanocomposite.

Wavelength/nm	t_1_/min	t_2_/min	A_1_	A_2_	R^2^
470	8.8	48.1	42,980	10,797	0.9994
660	8.9	48.8	111,110	26,721	0.9993
